# Clinical impact of drug-drug interactions on abemaciclib in the real-world experience of AB-ITALY study

**DOI:** 10.1038/s41523-024-00657-z

**Published:** 2024-07-17

**Authors:** Simone Scagnoli, Simona Pisegna, Angela Toss, Roberta Caputo, Michelino De Laurentiis, Michela Palleschi, Ugo de Giorgi, Enrico Cortesi, Agnese Fabbri, Alessandra Fabi, Ida Paris, Armando Orlandi, Giuseppe Curigliano, Carmen Criscitiello, Ornella Garrone, Gianluca Tomasello, Giuliana D’Auria, Patrizia Vici, Enrico Ricevuto, Federica Domati, Claudia Piombino, Sara Parola, Roberta Scafetta, Alessio Cirillo, Beatrice Taurelli Salimbeni, Francesca Sofia Di Lisa, Lidia Strigari, Robert Preissner, Maurizio Simmaco, Daniele Santini, Paolo Marchetti, Andrea Botticelli

**Affiliations:** 1 Department of Radiological, Oncological and Pathological Science, “Sapienza” University of Rome, Rome, Italy; 2 Department of Experimental Medicine, Sapienza University, Rome, Italy; 3 Department of Oncology and Hematology, Azienda Ospedaliero-Universitaria di Modena, Modena, Italy; 4 Department of Medical and Surgical Sciences, University of Modena and Reggio Emilia, Modena, Italy; 5 Department of Breast and Thoracic Oncology, Division of Breast Medical Oncology, Istituto di Ricovero e Cura a Carattere Scientifico (IRCCS) Pascale, Naples, Italy; 6 IRCCS Istituto Romagnolo per lo Studio dei Tumori “Dino Amadori” IRST Meldola IT, Meldola, Italy; 7 UOC Belcolle Hospital, Viterbo, Italy; 8 Precision Medicine in Senology, Department of Women Child and Public Health, Fondazione Policlinico Universitario A. Gemelli IRCCS, Rome, Italy; 9 Precision Medicine in Senology, Scientific Directorate, Fondazione Policlinico Universitario A. Gemelli IRCCS, Rome, Italy; 10 Division of Gynecologic Oncology, Department of Woman and Child Health and Public Health, Fondazione Policlinico Universitario Agostino Gemelli IRCCS, Rome, Italy; 11 Fondazione Policlinico Universitario Agostino Gemelli IRCCS Comprehensive Cancer Center, Unit of Medical Oncology, Rome, Italy; 12 Division of New Drugs and Early Drug Development for Innovative Therapies, European Institute of Oncology, IRCCS, Milan, Italy; 13 Department of Oncology and Hematology (DIPO), University of Milan, Milan, Italy; 14 Fondazione IRCCS Ca’ Granda Ospedale Maggiore Policlinico, SC Oncologia Medica, Milan, Italy; 15 Sandro Pertini Hospital Unit of Medical Oncology, Rome, Italy; 16 Istituto di Ricovero e Cura a Carattere Scientifico (IRCCS) Istituto Nazionale Tumori Regina Elena, UOSD Sperimentazioni di fase IV IT, Rome, Italy; 17 University of L’Aquila, L’Aquila, Italy; 18 Università Campus Biomedico, Rome, Italy; 19 IRCSS AOU Bologna, Bologna, Italy; 20 Institute of Physiology and Science-IT, Charité-Universitätsmedizin Berlin, Berlin, Germany; 21 Laboratory of Clinical Biochemistry, Sant’Andrea University Hospital, Rome, Italy; 22 Department of Neurosciences, Mental Health and Sensory Organs (NESMOS), Sapienza University of Rome, Rome, Italy; 23 Department of Medico-Surgical Sciences and Biotechnologies, Sapienza University of Rome, Rome, Italy; 24 Medical Oncology A, AOU Policlinico Umberto I, Rome, Italy; 25 Istituto Dermopatico dell’Immacolata IRCCS, Rome, Italy

**Keywords:** Breast cancer, Tumour biomarkers, Targeted therapies, Drug regulation

## Abstract

Abemaciclib demonstrated clinical benefit in women affected by HR+/HER2− advanced breast cancer (aBC). Drug-drug interactions (DDIs) can lead to reduced treatment efficacy or increased toxicity. This retro-prospective study aimed to evaluate outcomes, DDIs’ impact, and toxicities of abemaciclib combined with endocrine therapy in a real-world setting. Patients from 12 referral Italian hospitals with HR+/HER2− aBC who received abemaciclib were included. Clinical data about comorbidities, concurrent medications, outcomes, and adverse events (AE) were collected. Drug-PIN® (Personalized Interactions Network) is a tool recognizing the role of multiple interactions between active and/or pro-drug forms combined with biochemical and demographic patient data. The software was used to define the Drug-PIN score and Drug-PIN tier (green, yellow, dark yellow, and red) for each patient. Univariate and multivariate analyses were performed to identify predictors of patients’ PFS or toxicity. One hundred seventy-three patients were included. 13% of patients had >75years. The overall response rate (ORR) was 63%. The general population’s median PFS (mPFS) was 22 months (mo), while mOS were not reached. Patients treated with abemaciclib in combination with AI and fulvestrant had a mPFS of 36 and 19 mo, respectively. The most common toxicities were diarrhea, asthenia, and neutropenia detected in 63%,49%, and 49% of patients. The number of concomitant medications and comorbidities were not associated with survival outcomes (22 vs 17 mo, *p* = 0.068, *p* = 0.99). Drug-PIN tier from dark yellow to red and Drug-PIN score >12 were associated with shorter PFS compared to no/low-risk DDIs and score <12 (15 vs 23, *p* = 0.005, *p* = 0.0017). Drug interaction was confirmed as an independent biomarker in a multivariate model (*p* = 0.02). No difference in any grade AE, severe toxicities, and diarrhea were detected among different age subgroups. No association was found between Drug-PIN score or Drug-PIN tier and overall toxicity (*p* = 0.44), severe AEs (*p* = 0.11), or drug reduction (*p* = 0.27). The efficacy and safety of abemaciclib plus ET were confirmed in a real-world setting, even in the elderly population and patients with comorbidities. Evaluation of DDIs with Drug-PIN appears to be an independent predictor of PFS.

## Introduction

The discovery of cyclin-dependent kinases 4/6 (CDK4/6) inhibitors significantly reshaped the therapeutic landscape of hormone-receptor (HR)-positive, human epidermal growth factor receptor 2 (HER2)-negative advanced breast cancer (aBC)^1^. Among them, abemaciclib showed statistically significant and clinically meaningful improvements in progression-free survival (PFS) and overall survival (OS) while maintaining the health-related quality of life (HRQoL), both when given with fulvestrant in endocrine-resistant aBC and when given with a non-steroidal aromatase inhibitor (AI) in endocrine-sensitive aBC. Though this meaningful increase in clinical outcomes, final overall survival results for patients who received abemaciclib + AI as upfront treatment are still awaited^2–6^. Furthermore, abemaciclib showed improved invasive disease-free survival (iDFS) in patients with HR+/HER2−, high-risk early breast cancer (eBC) combined with standard-of-care adjuvant endocrine therapy after surgery^7^.

An age-specific subgroup analysis of patients older than 65 years enrolled in MONARCH-2 and MONARCH-3 trials showed higher rates of nausea, decreased appetite, and venous thromboembolic events^8^. Since a significant share of HR+/HER2− aBC occur in older women with high rates of comorbidities and concomitant medications, post-marketing surveillance studies and real-world studies are pivotal to confirming the safety and the efficacy of abemaciclib in a wider, unselected populations^9,10^.

The increasing use of CDK4/6i in HR+/HER2− aBC in clinical practice has rekindled the interest in drug–drug interactions (DDIs) since interactions cause one drug to affect the other drugs and can lead to a reduced treatment efficacy or increased toxicity, with a potential effect on clinical outcomes^11,12^.

Pharmacokinetics and pharmacodynamics-based DDIs in cancer patients taking multiple concomitant medications may alter anti-cancer drugs’ therapeutic index, thus causing reduced compliance, unwanted adverse drug reactions (ADRs), and treatment failure^13,14^. Despite this relevant clinical issue, few retrospective analyses have been conducted to evaluate DDIs in patients receiving anti-cancer treatments, and among these, study populations were widely heterogeneous. Moreover, most patients were treated with intravenous chemotherapy, and no data were available for patients with aBC treated with CDK4/6 inhibitors^15,16^.

The liver primarily metabolizes Abemaciclib through various cytochrome P450 (CYP) enzymes. The main enzymes involved in its metabolism are CYP3A4 and, to a lesser extent, CYP2C9. Abemaciclib itself is considered the major active component. The primary metabolites formed during its metabolism, M2 and M20, have shown minimal pharmacological activity in preclinical studies. Therefore, the therapeutic effects are primarily attributed to the drug itself. Abemaciclib and its primary active metabolites inhibit renal transporters, such as the transporter of organic cation 2 (OCT2), the multidrug and toxin extrusion protein (MATE1), and the MATE2-K. In vivo, interactions may occur with relevant substrates of these carriers, such as creatinine and metformin^17^.

Patients on abemaciclib may suffer from clinically relevant drug interactions causing altered drug exposure; patients with multiple comorbidities and the elderly could be the higher-risk population.

Therefore, it is evident that the analysis of multiple layers of data from the same patients can be critical in shaping clinical outcomes.

In this multicentre, retrospective study, we aimed to investigate the impact of poly-pharmacotherapy and drug interactions in a real-world cohort of patients with either endocrine-resistant or endocrine-sensitive aBC treated with abemaciclib plus standard endocrine therapy (ET).

## Results

**Fig. 1:**
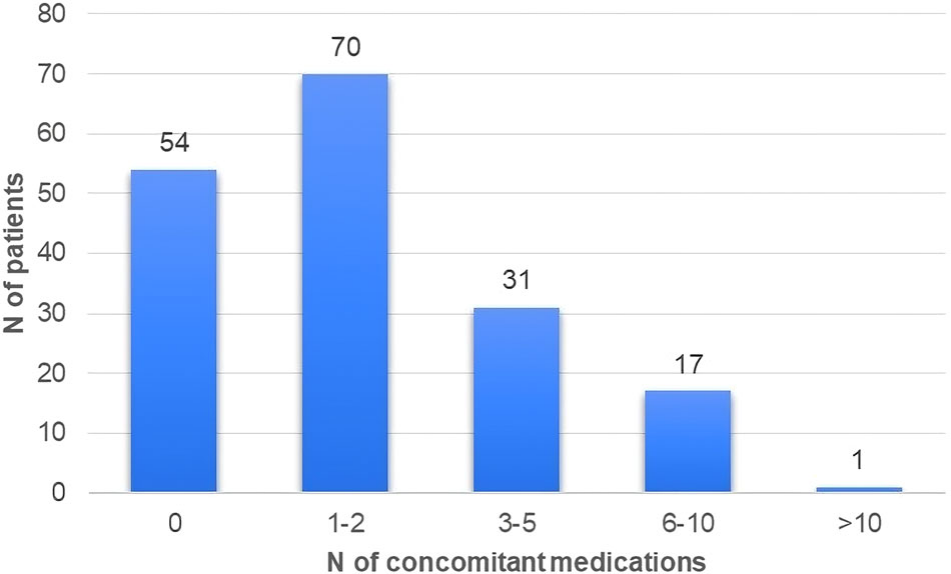
Concomitant medications in the study population. Number of concomitant medications divided into five groups of patients. On the *x*-axis is reported the number of concomitant medications. On the *y*-axis is reported the number of patients. The cumulative number of patients per group is represented by the height of the histogram and reported at the top of the columns.

One hundred seventy-three (173) patients were retrospectively included. Patients’ characteristics are reported in Table 1. The median age was 60 years (range 37–87). Sixty-eight patients (39%) were 65 years old or older, with 23 (13%) over 75 years old. The majority of patients presented a postmenopausal status (59%). Globally, 106 (61%) patients had at least one comorbidity, and 23 (13%) had two or more. The remaining reported no significant comorbidities. 40% of patients were receiving 1–2 concomitant medications, and 18 (10%) assumed more than five medications per day (Fig. 1). Median Drug-PIN score was 3.25 (range 0–114). Moreover, 149, 7, 14, and 3 patients were in the green, yellow, dark yellow and red tier, respectively. According to this result, 17 patients (10%) fall into the dark yellow and red categories, with potentially intermediate and high-risk drug interactions detected in 14 and 3 patients, respectively. Patients’ characteristics according to the DDI risk are reported in Table 2. Median Drug-PIN score and Drug-PIN tiers remain similar when adding abemaciclib to the patients’ concomitant therapy (3.25 vs. 4.9, *p* = 0.44), indicating that DDIs are mostly related to concomitant medications. Abemaciclib was received as the upfront treatment in 144 (80%) patients. Globally, abemaciclib was administered in combination with AI or fulvestrant in 81 (47%) and 92 (53%) patients, respectively. In the context of the first line, 53% of patients were endocrine-sensitive and received abemaciclib in combination with AI, while 47% were endocrine-resistant and treated with abemaciclib plus fulvestrant. Seventy-six (44%) patients had visceral disease, 35 had liver metastases.

**Table 1:** Patients’ characteristics

Characteristics	*N* (%)
*Age*	
Median, years (range)	60 (37–87)
>60 years	85 (49)
≤60 years	88 (51)
*Menopausal status*	
Premenopausal	69 (40)
Postmenopausal	103 (59)
Other/men	1 (1)
*Comorbidities*	
Any	67 (39)
None	106 (61)
*Comorbidities, number*	
0	67 (39)
1–2	83 (48)
>2	23 (13)
*Concomitant medications*	
Median, no. (range)	1 (0–11)
*Concomitant medications, number*	
0	54 (31)
1–2	70 (40)
3–5	31 (18)
6–10	17 (10)
>10	1 (0)
*Advanced disese, timing*	
Synchronous	42 (24)
Metachronous	131 (76)
*Endocrine therapy partner*	
NSAI	81 (47)
fulvestrant	92 (53)
*Visceral disease*	
Yes	76 (44)
No	*97(56)*

*N* number of patients, *NSAI* nonsteroidal aromatase inhibitors.

**Table 2:** Patients’ characteristics according to DDIs risk

Characteristics	No-low risk DDIs *N* (%)	Intermediate-high risk DDIs *N* (%)	*p*
*Age*			
≤60	83 (53)	5 (30)	
>60	73 (47)	12 (70)	0.06
*Menopausal status*			
Premenopausal	63 (41)	6 (35)	
postmenopausal	92 (59)	11 (65)	0.87
*Comorbidities*			
None	63 (41)	5 (30)	
Any	92 (59)	12 (70)	0.15
*Comorbidities, number*			
0	65 (42)	2 (12)	
1–2	76 (48)	7 (42)	
>2	15 (10)	8 (46)	**0.01**
*Concomitant medications, number*			
1–2	70 (68)	0 (0)	
3–5	26 (25)	5 (30)	
6–10	6 (7)	11 (65)	
>10	0 (0)	1 (5)	**<0.01**
*Endocrine therapy partner*			
NSAI	72 (46)	9 (53)	
fulvestrant	84 (54)	8 (47)	0.78

*N* number of patients, *NSAI* nonsteroidal aromatase inhibitors, no-low risk DDIs: tiers green and yellow; intermediate-high risk DDIs: tiers dark yellow and red; in bold: *p* value < 0.05.

### The efficacy of Abemaciclib plus ET was confirmed in a real-world population

**Fig. 2:**
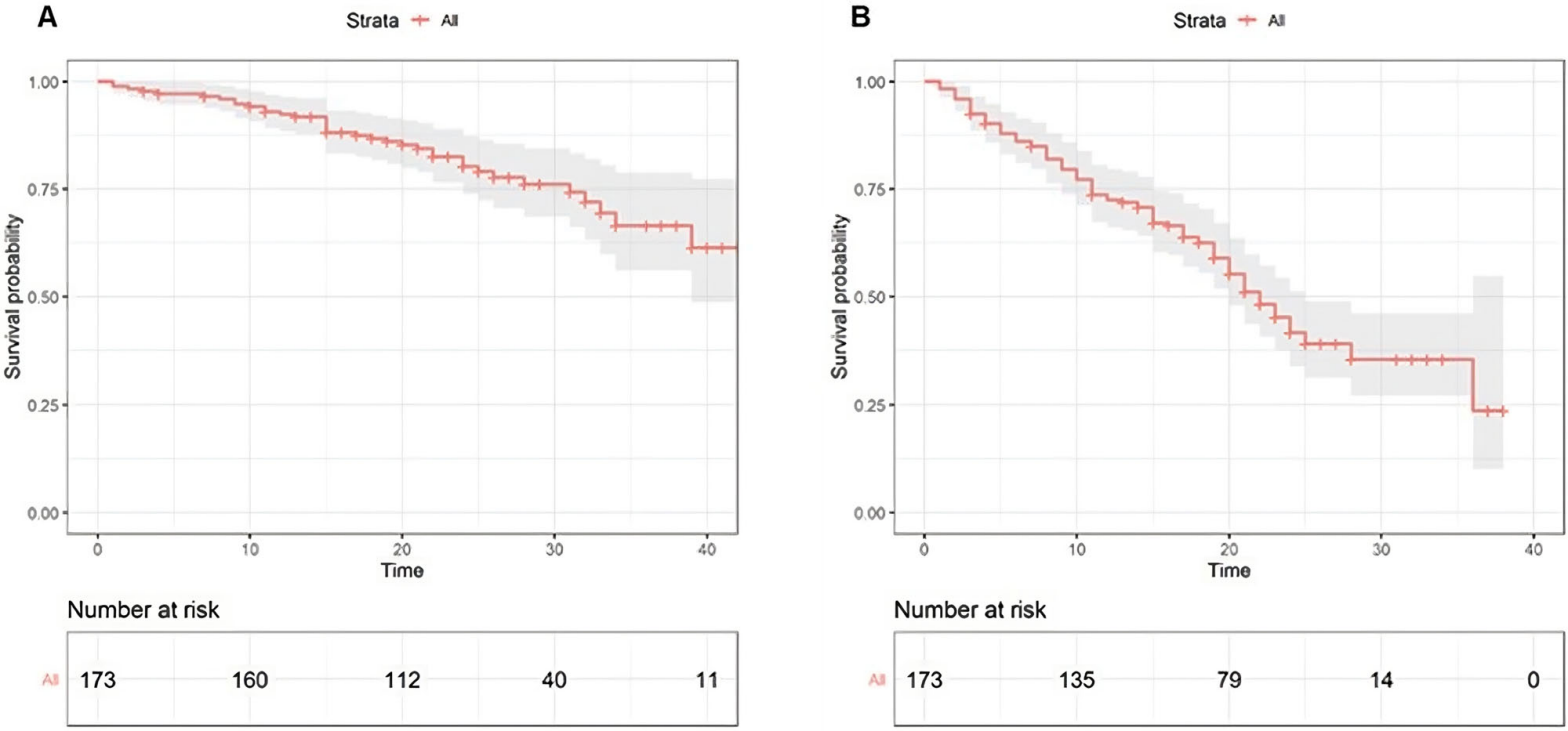
OS and PFS in the study population. Kaplan–Meier estimates of OS (**A**) and PFS (**B**) in the overall population (red lines). mOS was not reached. mPFS was 22 (range) months. The gray area represents the confidence interval. Tick marks represent data censored at the last time the patient was known to be alive.

Among patients with measurable disease (156), the overall response rate (ORR) was 63%, and the clinical benefit rate (CBR) was 92%. The best response was complete response (CR) or partial response (PR) in 6 (4%) and 92 (59%) patients, respectively; stable disease (SD) and progressive disease (PD) were reported in 46 (29%), and 12 (8%) patients, respectively (Suppl. Table 2). At a median follow-up of 30 months (11–46), the median PFS (mPFS) in our population was 22 months (range 1–37) (Fig. 2 B).

**Fig. 3:**
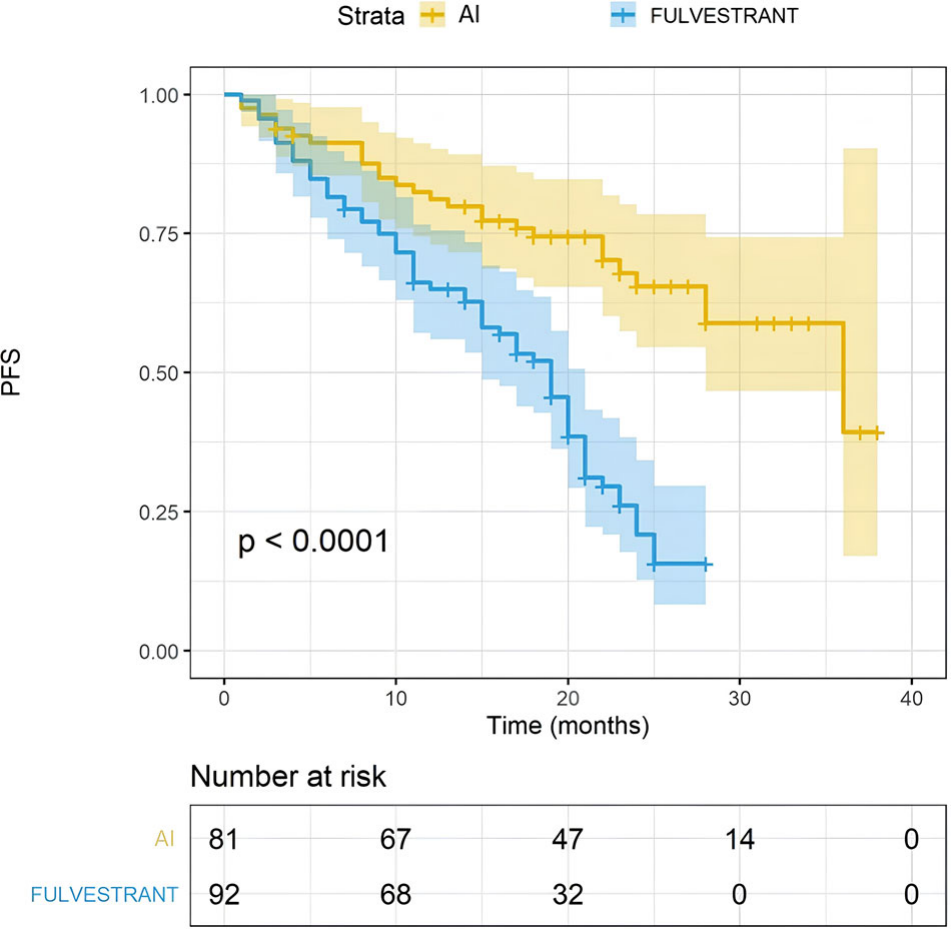
FS according to abemaciclib companion. Kaplan–Meier estimates of PFS, according to the combination of abemaciclib + Aromatase Inhibitor (yellow line) or abemaciclib + fulvestrant(blue line); 35 months vs 19 months, *p* < 0.0001). The colored area represents the confidence interval. Tick marks represent data censored at the last time the patient was known to be alive.

Firstly, we evaluated the impact of the main clinical features on PFS. Patients treated with upfront abemaciclib + AI had a PFS of 36 months compared to 19 months of the combination abemaciclib + fulvestrant, administered in the endocrine-resistant population (Fig. 3; *p* < 0.0001). Patients with endocrine sensitivity had a longer PFS compared to endocrine resistance (*p* = 0.003; Suppl. Fig. 1). The number of comorbidities did not seem to impact clinical outcomes (Suppl. Figs. 2 and 3; *p* = 0.998; *p* = 0.766). We grouped patients by age in <65 y, 65 y to 70 y, 71 y to 74 y, and ≥75 y. An age ≥75 y was associated with a shorter PFS if compared to patients with less than 65 y (Suppl. Fig. 4, 18 vs. 25 months, *p* = 0.009), while there was no significant difference among the other age-based groups. Patients with visceral disease have a trend in shorter PFS compared to those without (Suppl. Fig. 5, 23 vs. 19 months, *p* = 0.063). Liver disease only was not associated to worse PFS (*p* = 0.09).

### High DDIs were associated with worse PFS

**Fig. 4:**
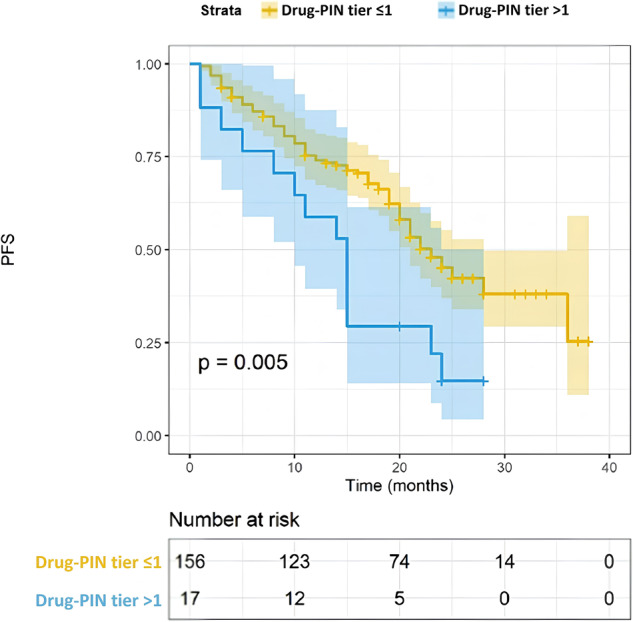
PFS according to polypharmacy. Kaplan–Meier estimates of PFS, according to polypharmacotherapy (no polipharmacotherapy, yellow line; yes polypharmacotherapy, blue line any grade; 22 months vs 17 months, *p* = 0.068). The colored area represents the confidence interval. Tick marks represent data censored at the last time the patient was known to be alive.

**Fig. 5:**
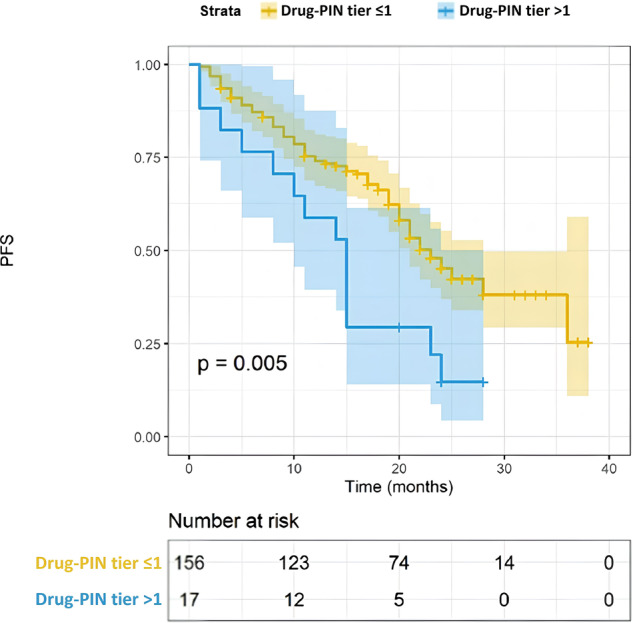
PFS according to Drug-PIN tier. Kaplan–Meier estimates of PFS, according to Drug-PIN tier (≦1 low-risk interactions, yellow line; >1, high-risk interactions; 23 vs 15 months, *p* = 0.005). The colored area represents the confidence interval. Tick marks represent data censored at the last time the patient was known to be alive.

**Fig. 6:**
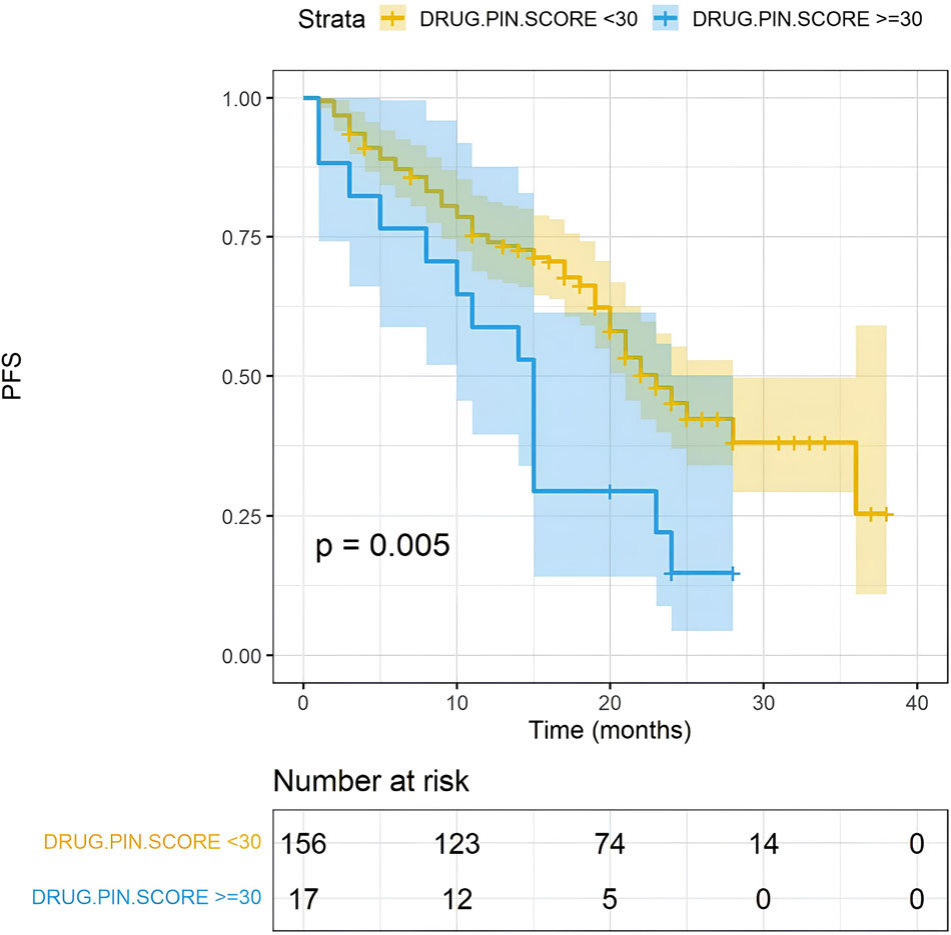
PFS according to Drug-PIN Score. Shown are Kaplan–Meier estimates of PFS, according to Drug-PIN score (<30, yellow line; ≥30, blue line any grade; 23 vs 15 months, *p* = 0.005). The colored area represents the confidence interval. Tick marks represent data censored at the last time the patient was known to be alive.

**Fig. 7:**
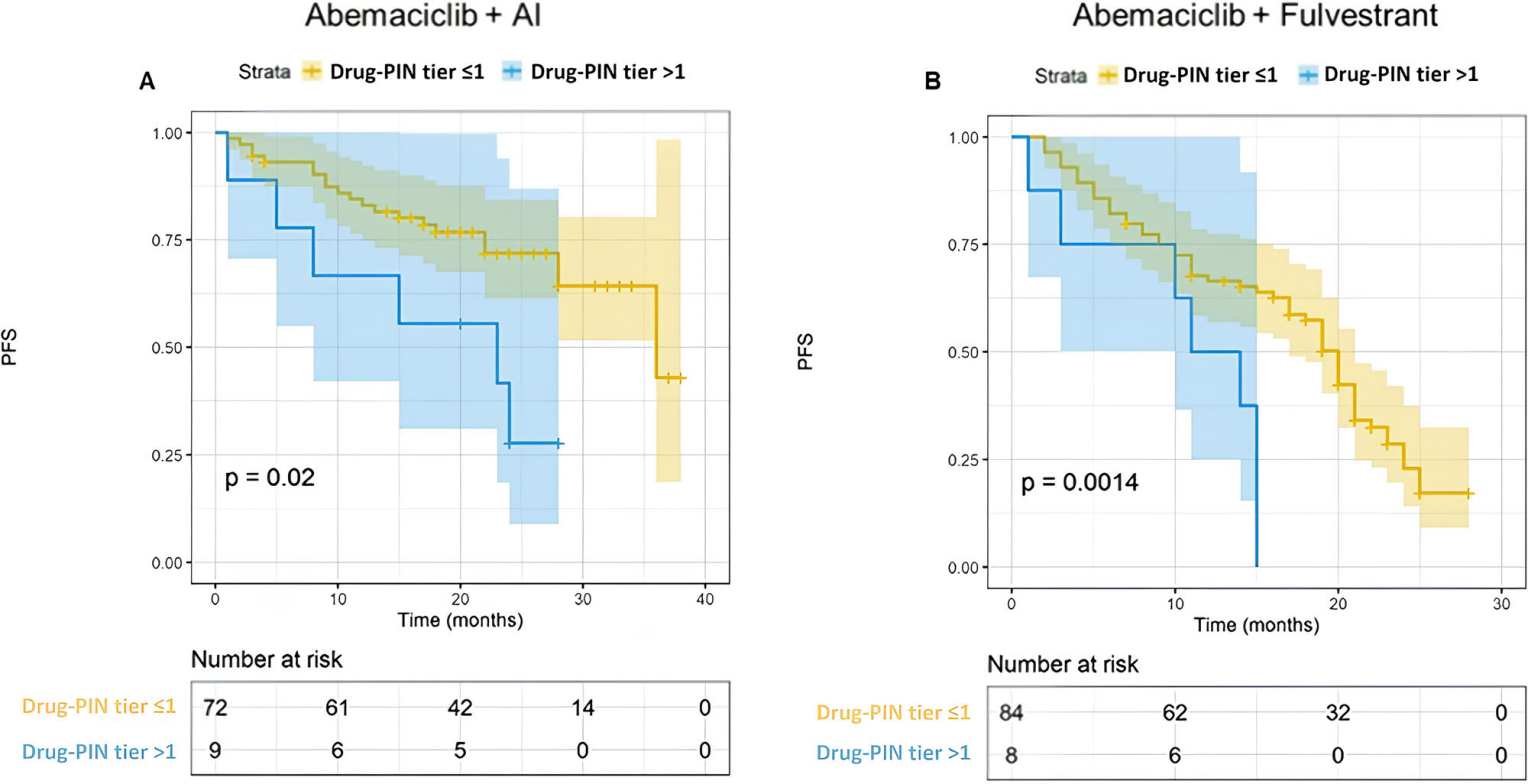
PFS according to Drug-PIN tier in different settings. Shown are Kaplan–Meier estimates of PFS, according to Drug Pin tier (≦1 low-risk interaction, yellow line; >1, high-risk interactions) in the two different populations of patients treated with upfront abemaciclib + AI (**A**; *p* = 0.02) or abemaciclib + fulvestrant (**B**; *p* = 0.0014). PFS was significantly worse in patients with high-risk DDIs compared to patients with low-risk risk DDIs, regardless of the treatment received. The colored area represents the confidence interval. Tick marks represent data censored at the last time the patient was known to be alive.

Furthermore, we focused on the impact of concomitant medication and the consequent DDIs. Polypharmacy and the number of concomitant drugs assumed by the patients did not significantly impact PFS (Fig. 4, 22 vs 17 months, *p* = 0.068). However, the identification of high DDIs was significantly associated with a worse PFS. Patients with a Drug-PIN tier indicating a high risk of interaction had a PFS of 15 months compared to 23 months of those with low-risk interactions (Fig. 5, *p* = 0.005). A similar result was obtained by evaluating the Drug-PIN score (Fig. 6, *p* = 0.005). Moreover, to avoid a possible selection bias, we assessed the effect of DDIs in both the abemaciclib + AI and abemaciclib + fulvestrant populations. PFS was significantly worse in patients with high-risk DDIs compared to patients with low-risk DDIs, regardless of treatment received (Fig. 7A, B; *p* = 0.02 and *p* = 0.0014). Altogether, using a UVA, the association with AI or Fulvestrant (*p* < 0.0001), the age (*p* < 0.009), and the Drug-PIN tier (high-risk interactions, from dark yellow to red, *p* = 0.005) were significant predictive factors of decreased PFS. Finally, using a multivariate Cox regression model, a significant association was shown between Drug-PIN tier and PFS, confirming the high DDIs risk as an independent predictive factor of worse PFS (HR 2.214, 95% CI 1.209–4.149; *p* = 0.013; Suppl. Fig. 6).

Overall survival data are still immature, and the entire population’s median Overall Survival (mOS) has still not been reached (Fig. 2A). However, in patients with high-risk DDIs, there is a trend in shorter OS compared to the rest of the population (Suppl. Fig. 7).

### Abemaciclib toxicity had no impact on survival outcomes

**Fig. 8:**
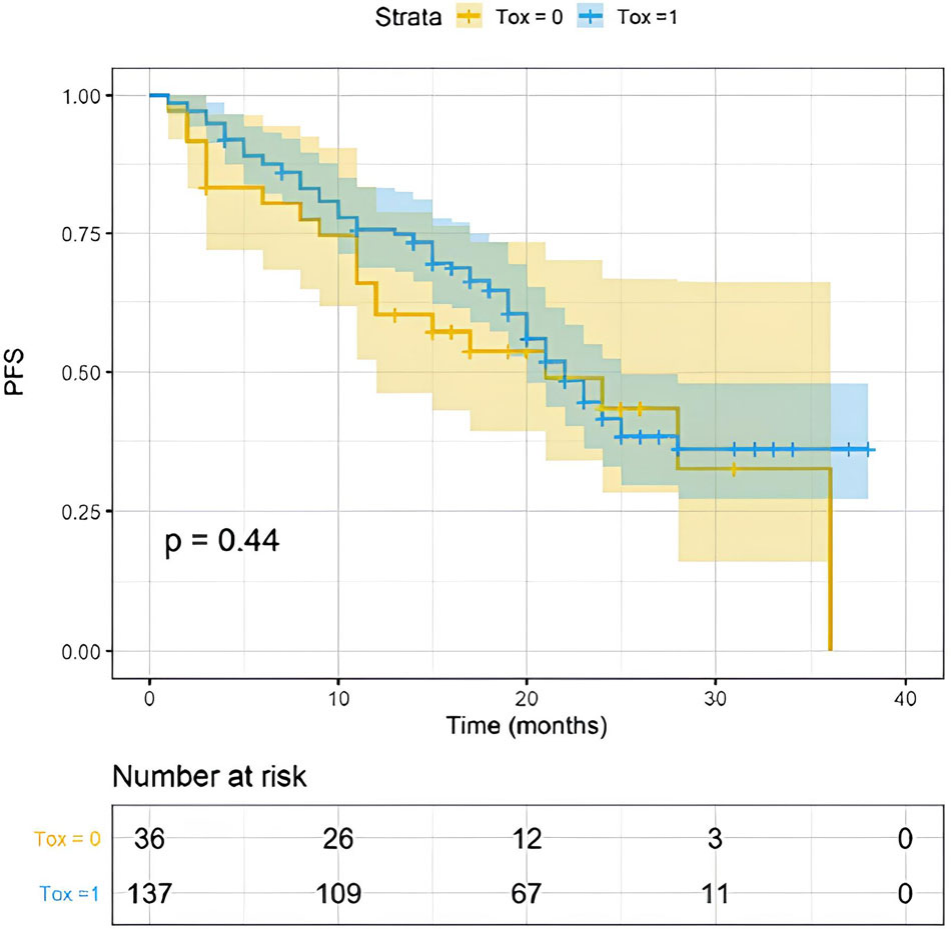
PFS according to treatment toxicity. Shown are Kaplan–Meier estimates of PFS, according to toxicity group (0 = no toxicity, yellow line; 1 = at least one toxicity of any grade, blue line any grade; 21 vs 22 months, *p* = 0.44). The colored area represents the confidence interval. Tick marks represent data censored at the last time the patient was known to be alive.

Treatment-related toxicities are summarized in Table 3. Any grade adverse events (AEs) were registered in 133 (77%) patients. Thirty-one (18%) patients experienced a severe AE (G3/G4). Diarrhea was the most common toxicity reported in 109 patients (63%; any grade). However, severe diarrhea was experienced only by 4% of patients. The onset time was the first month/cycle for most patients (median one month; range 1–17) and lasted one week or less for 48% of patients (median one week; range 1–40 weeks). Other common AEs were asthenia (85 pts; 49% any grade) and neutropenia (85 pts; 49% any grade). Anemia was registered in 62 patients (36%; any grade). The most common G3/G4 toxicities were neutropenia, diarrhea, anemia, and asthenia observed respectively in 18, 8, 5, and 4 patients. Deep vein thrombosis (DVT)/pulmonary embolism was registered in 3 patients (1.7%). A dose reduction was required in 32% of patients, and 10% discontinued due to toxicity. AEs were similar in young and elderly patients. In particular, no difference in AEs of any grade, severe AEs, and diarrhea was detected among patients aged ≥65 y and ≥75 y compared to younger ones (Table 4). Menopausal status and comorbidities were not related to the occurrence of AEs. The use of fulvestrant was associated with a higher toxicity rate (AUC ROC of Fulvestrant vs AI for overall AEs 0.602; 95% CI = 0.525 to 0.675; *p*-value = 0.0373). Drug-PIN score or Drug-PIN tier were not associated with overall toxicity (*p* = 0.44), severe AEs (*p* = 0.11) or dose reduction (*p* = 0.27). Using a UVA, DDIs were associated as a trend with severe diarrhea, DVT, and anemia of any grade (Suppl. Table 3). AEs do not seem to impact PFS (Fig. 8, *p* = 0.44) and OS (Suppl. Fig. 8, *p* = 0.65). Finally, patients who reduced the dose of abemaciclib due to AEs achieved similar PFS to those who did not (Suppl. Fig 9, *p* = 0.54).

**Table 3:** Treatment-related adverse events

Adverse events	*N* (%)
*Adverse event, any*	
Yes	133 (77)
No	40 (23)
*Dose reduction, any*	
Yes	56 (32)
No	117 (68)
*Diarrhea*	
Any grade	109 (62)
Grade 3/4	8 (4)
*Neutropenia*	
Any grade	85 (49)
Grade 3/4	18 (10)
*Ashtenia*	
Any grade	85 (49)
Grade 3/4	4 (2)
*Anemia*	
Any grade	62 (36)
Grade 3/4	5 (3)
*Nausea*	
Any grade	15 (8)
Grade 3/4	1 (0)
*Hepatotoxicity*	
Any grade	14 (7)
Grade 3/4	3 (1)
*Renal toxicity*	
Any grade	35 (19)
Grade 3/4	1 (0)
*DVT/pulmunary embolism*	
Yes	6 (3)
No	167 (97)

*N* number of patients, *DVT* deep vein thrombosis.

**Table 4:** AEs per age category

Adverse events	Age category		*p*	Age category		*p*
	<65	≥65		<75	≥75	
Any grade AEs	79%	79%	0.95	79%	82%	0.66
Severe AEs	15%	23%	0.17	17%	26%	0.31
Diarrhea	67%	61%	0.43	67%	52%	0.15
Severe diarrhea	6%	6%	0.96	5%	8%	0.51

*AEs* adverse events; severe: grade 3 and 4 according to CTCAE v5.

## Discussion

In HR+/HER2− aBC, CDK4/6 inhibitors plus ET have become the current standard of care as first or second-line treatment, giving impressive clinical benefits in both endocrine-sensitive and endocrine-resistant diseases^3,5,18–21^.

In our study, we retrospectively investigated the efficacy and toxicity profile of abemaciclib in a real-world setting of HR+/HER2− aBC patients. In addition, we also assess DDIs and their potential impact on clinical outcomes.

Abemaciclib appeared to be effective and safe in our unselected population, as well as in older patients and patients with comorbidities. Sixty-five years is commonly considered as the main age-related cut-off^22,23^. In our real-world population, there is a high rate of ≥ 65-year-old patients (40%), with several patients aged >75 years old (13%). Abemaciclib was safe in this particular population, with a similar number and grade of AEs in the elderly compared to younger patients. Moreover, no difference in PFS was detected between patients aged ≥65 y and <65 y old. However, if we consider the small subgroup of patients ≥75 y, they had a worse PFS than younger ones. This data agrees with the known literature as age ≥75–80 years is a known adverse prognostic factor, and often, these patients have worse baseline general conditions^24–26^. Anyway, the sample size is limited, and a specific prospective, real-world study should be conducted in this particular population.

In this real-world study, the efficacy of abemaciclib was comparable to clinical trials. We found an ORR of 63%, similar to what has been reported in registration trials (about 50–60%)^3,5^. The high ORR confirms the relevant tumor shrinkage determined by abemaciclib plus ET, which makes the combination useful even in conditions of high tumor burden.

Regarding the survival outcomes, PFS in the overall population was 22 months, while the median OS was still not reached. As expected, PFS was longer in patients treated in the endocrine-sensitive setting with abemaciclib plus AI (mPFS 35 months) as compared to patients treated with abemaciclib plus fulvestrant in the endocrine-resistant setting (mPFS 19 months). In particular, in both subgroups, the real-world PFS is comparable and consistent with the respective registrative clinical trials (i.e., Monarch 3 (abemaciclib + AI; mPFS 29 months) and Monarch 2 (abemaciclib + fulvestrant; mPFS 17 months)^3,27^. This crucial data confirms the efficacy of abemaciclib in combination with AI and fulvestrant in an unselected, real-world population.

The median follow-up of our study was 30 months. This observation period is long enough to evaluate PFS but is relatively short for OS. The expected mOS in patients with HR+/HER2− aBC who receive CDK4/6i as the first line may range from 50 to 65 months^28^. As expected, the mOS of our population is not reached (Fig. 2). Anyway, we consider the known limitations of a real-life study, especially on the assessment of disease outcomes such as non-uniformity among different centers in detecting the exact time of progression, different timing of radiological re-evaluation, non-centralization of radiological imaging assessments and possible delays in defining progressions or knowing the exact death of the patient.

Reported AEs were also consistent with known literature, without any signal of a different safety profile^8^. Toxicities of any grade were reported in 77% of patients, with 18% of severe AEs. No treatment-related death was reported. As expected, diarrhea was the most frequent AE (63%). The rate of severe diarrhea was very low (4%), and the duration of diarrhea was short (1 week), confirming the manageability of this AE even in the real-world population. DVT was a rare adverse event (<2%), and no interstitial lung disease (ILD) was reported. Elderly patients had a similar toxicity profile to younger patients, and no differences in discontinuation rate were detected. Our study adds important information on the safety of abemaciclib in this particular population, which is underrepresented in clinical trials.

Polypharmacy was confirmed as a relevant clinical issue, with 119/173 (69%), 49/173 (28%), and 18/173 (10%) patients taking >1, >3, and >5 drugs every day, respectively.

Hepatic metabolism primarily by cytochrome P450 (CYP) 3A4 is the main route of clearance for abemaciclib; therefore, abemaciclib’s pharmacokinetics are significantly impacted by CYP3A4 inhibitors and inducers^29–31^. Moreover, when patients take many concomitant drugs, the DDI can be complex and involve both pharmacokinetic and pharmacodynamic mechanisms^32–35^. This complex system of interactions can be barely predicted and modulated by a single specialist as the oncologist without dedicated tools. Drug-PIN is a software that combines clinical data (age, weight, liver, and renal function) with the DDIs and, if available, the pharmacogenomics profile^36^. The output of the Drug-PIN system indicates how high is the risk of interactions and if the concomitant drugs have to be changed in a *drug reconciliation* process. This software allows reaching a highly tailored approach for every patient. Even if most patients had no to low-risk interactions in our real-world population, as highlighted by the median Drug-PIN score of 3.25, about 10% of patients had a potentially dangerous drug interaction. This rate is in line with data coming from another large study^37,38^. However, we calculated the Drug-PIN output before and after the introduction of abemaciclib in the routine treatment of the patients, and no significant difference was detected in the overall median score. Indeed, adding abemaciclib to the baseline medications did not affect the score in most patients, confirming that abemaciclib is characterized by a low risk of DDIs. LHRH agonists were included in the DDIs evaluation for all premenopausal patients. Triptorelin might interfere with some drugs used to treat arrhythmias (e.g., quinidine, procainamide, amiodarone, and sotalol) or might increase the risk of arrhythmia when taken with methadone moxifloxacin and antipsychotics while leuprorelin has no interactions. Among the 71 premenopausal patients in our study, none had a relevant interaction due to the LHRH agonist. As expected, no change in DDIs score were detected from the addition of letrozole or fulvestrant to concomitant medications. As expected, patients with more comorbidities and with a greater number of drugs on therapy are more frequent in the group with high-risk DDIs (Table 2)^39^. However, neither the number of comorbidities nor the number of medications taken significantly impact PFS in our population.

The survival analysis showed that patients with high Drug-PIN scores and tier had significantly worse PFS than patients with no/low DDIs (23 vs. 15 months; *p* = 0.005). As defined in the methods, we found the Drug-PIN score “30” as the cut-off that reaches the most significant *p*-value in our population (Fig. 6). We found “12” as the cut-off closest to the median Drug-PIN score that identifies patients with reduced PFS (Suppl. Fig. 10). However, this value, which affects about 20% of the population, must be studied in future validation cohorts. Conversely, it is relevant to note that the number of concomitant drugs and the polypharmacy were not associated with survival outcomes. This emphasizes the importance of the quality of drug interactions over the sheer quantity of medications the patient takes. Considering the expected different PFS among patients treated with abemaciclib + AI vs. abemaciclib + fulvestrant, we evaluated the DDIs separately for the two groups. The predictive value of Drug-PIN was confirmed in both endocrine-sensitive and endocrine-resistant settings: patients with high-risk DDIs had a shorter PFS regardless of the association with AI or fulvestrant. Patients who received fulvestrant were all pretreated or progressed during adjuvant therapy. Therefore, Drug-PIN seems to predict worse PFS whether or not the patients have received prior treatments. Considering the visceral disease’s clinical relevance, we added this variable to our multivariable Cox model, even if our population’s results showed a trend (*p* = 0.063) without a significant *p*-value using the univariate analysis. Finally, the MVA confirmed Drug-PIN as an independent predictive factor of PFS (*p* = 0.013), together with age, ET association, and visceral disease. Furthermore, we demonstrated that the multicollinearity is not an issue for the MVA of PFS in our dataset (Suppl. Table 4). With all the limitations of a retrospective/prospective real-world study and a limited sample size, the results obtained may lay the foundations for future studies on the usefulness of advanced DDI evaluation software and the effect of DDIs on cancer treatments. The reduced efficacy could be due to an unexpected metabolic alteration or a greater frailty of the patient with DDIs. Given the retrospective nature of this study, the finding could also be related to other unevaluated clinical and pathological features. Further investigations are needed to clarify this point. An additional limitation involves the assessment of DDIs with the patient’s baseline concomitant therapy. Patients may undergo changes in their medication regimens after the initiation of abemaciclib, even if no significant modifications are documented in the database. Prospective data, incorporating adjustments in concomitant medication from baseline, are essential for conducting a dynamic assessment of the effects of drug interactions. Moreover, considering the limited number of patients in the high-risk DDIs group (10%), the results must be cautiously interpreted and confirmed on a larger population.

However, no significant difference in the number or severity of AEs was reported in patients with or without high DDIs. We found only a numerically higher number of severe diarrhea (*p* = 0.07), DVT (*p* = 0.09), and anemia of any grade (*p* = 0.06) in patients with high Drug-PIN scores. These trends would probably be more significant by expanding the sample size.

We found a reduction in efficacy in terms of PFS but not an increase in toxicities in patients with severe DDIs treated with abemaciclib. Several papers and international guidelines reported that drug interactions can cause a reduction in efficacy, if not a failure, of medical treatments. In our population, interactions could mostly lead to decreased drug concentration and distribution or hinder drug activity. Evaluation of serum drug concentration could be helpful to confirm this hypothesis. However, this procedure is generally expensive, complex, and challenging to implement in clinical practice and was not foreseen in our real-world study. Furthermore, changes in serum concentration outside the therapeutic range may only partially explain the effect of DDis. The use of advanced AI-driven network tools such as Drug-PIN (see Data Availability Statement) can accelerate the assessment of DDIs, making it compatible with daily clinical practice and helping clinicians in the decision-making process. In addition, these tools can intercept multiple drug interferences that would be complex to assess with single analyses. Understanding the pharmacodynamics and pharmacokinetic interactions in the context of the individual patient’s characteristics, such as age, body conformation, liver and kidney function, diet, and habits, represents a crucial challenge in the future of precision medicine^13,40–45^.

In conclusion, we confirmed that abemaciclib is safe and effective in a real-world population, as well as in an elderly population and patients with comorbidities. DDIs evaluation seems to be an independent predictor of PFS. Further real-world, country-based studies with similar large populations are encouraged to support our results. Prospective studies with the addition of pharmacogenomic evaluations would help clarify the impact of DDIs on cancer treatments.

## Methods

### Study population and clinical endpoints

Clinical and pathological features of patients were collected from referral hospitals to treat aBC in Italy. ABC was defined as locoregionally recurrent breast cancer not amenable to surgical resection or radiotherapy with curative intent or metastatic disease. Patients with histological and radiological confirmation of HR+/HER2− aBC who received abemaciclib in combination with aromatase inhibitor (AI) or fulvestrant (F) as upfront or second-line treatment were included in the study. Endocrine sensitivity or resistance was defined according to ESMO guidelines^46^. According to guidelines, patients who received abemaciclib plus AI were all in the first-line setting and endocrine-sensitive. In contrast, patients receiving abemaciclib plus F progressed during or at the immediate end of adjuvant treatment in the early setting or progressed from a previous endocrine or chemotherapy first line in the metastatic setting^46^. Complete clinical data about comorbidities, concurrent medications, clinical outcomes of the treatment, and the completion of at least one month of abemaciclib were further inclusion criteria. Patients treated with a different CDK4/6i or affected by another malignancy were excluded. Visceral disease was defined as the presence of at least one metastatic lesion in the liver and/or lung and/or brain and/or peritoneum. Patients with only bone and/or lymph node and/or soft tissue metastases were included in the non-visceral group. The best response was evaluated following RECIST 1.1 criteria^47^. CTCAE v5 was used to assess treatment-related toxicities^48^. Overall response rate (ORR) was defined as the proportion of patients who achieved a partial or complete response.

### Drug–drug interactions assessment

Drug-PIN® was used to assess the pharmacological interaction between drugs^36^. Drug-PIN® (Personalized Interactions Network) is a medical software that can evaluate clinical, biochemical, and demographic patients’ data and combine them with a simultaneous DDIs profile, exploring possible effects between active and/or pro-drug forms^32,36,49^. The tool has been shown to improve prescription safety, facilitate the evaluation of DDIs in clinical practice, and identify patients at high risk of toxicity or reduced compliance with oncological and non-oncological treatments^50–53^.

Moreover, the software combines and analyses the clinical data (age, weight, liver, and renal function), the DDIs, and, if available, the genomic profile of patients. The output of the Drug-PIN system is a numerical score (Drug-PIN score) that indicates the risk of DDIs, and a tier (Drug-PIN tier: green, yellow, dark yellow, and red), which indicates how high the risk of interactions and if the concomitant drugs have to be changed, in a *drug reconciliation* process. The output ranges are divided as follows: Drug-PIN scores from 0 to 20 (green tier) define no interactions; from 20 to 30 (yellow tier) define low-risk DDIs; from 30 to 70 (dark yellow tier) defines intermediate-high risk DDIs; ≥70 (red tier) defines high-risk interactions. The following features were considered for each patient: age, race, smoking habit, alcohol consumption, and concomitant medications. The software performed a multi-pass analysis using machine learning algorithms, integrating data for each element added to the patient’s record. A specific drug-PIN score/tier was obtained for every patient without and with abemaciclib addition. The drug interaction test was performed with the concomitant medication reported at baseline.

### Statistical analysis

Univariate analysis (UVA) was conducted using the Kaplan and Meier curves of PFS and OS. The differences between the Kaplan–Meier curves were assessed using the log-rank test. At the UVA, the optimal cut-off of drug pin and tier score was determined by varying the cut-off and selecting the value minimizing the *p*-value of Kaplan–Meier curves of PFS. The multivariate Cox proportional hazard regression model (MVA) was implemented to assess the early predictor of patient clinical outcomes, while the logistic multivariate regression analysis was conducted to identify early predictors of toxicity or best response.

The treatment response/toxicity was used as the gold standard for non-parametric clustered ROC analysis to evaluate the predictive utility of the univariate or multivariate model. By comparing observed and calculated toxicity, the sensitivity and the specificity of each investigated variable or model were plotted using the receiver operating characteristic (ROC) curve and determining the 95% Confidence interval (CI). When a perfect correlation of predicted versus observed response was found, the area under the curve (AUC) was equal to 1, whereas random assignment of outcome led to a ROC/AUC of 0.5^54,55^.

Data were collected in an anonymous database and analyzed with R-package and SPSS v.26

### Ethical approval

The study was approved by the Institutional Review Board (IRB) of the coordinating center (Sapienza no. 0799/2020) and by the IRBs of each participating center (Suppl. Table 1). All procedures performed were in accordance with the Helsinki Declaration. Overall, 12 Italian centers adhered to the initiative. It is noteworthy that more participating centers referred to one single IRB. For deceased or unreachable patients, a waiver of consent was permitted by the ethics committee in accordance with the national regulation no. 72 published on 26 March 2012. All the patients who were alive at the time of the study approval signed a specifically conceived informed consent form.

### Reporting summary

Further information on research design is available in the Nature Research Reporting Summary linked to this article.

### Supplementary information

Supplemental material

reporting summary

## Data Availability

The datasets analyzed during the current study are available from the corresponding author on reasonable request. Please mail to simona.pisegna@uniroma1.it. Drug-PIN software licence is available for free upon specific request for clinical and research purpose at https://www.drug-pin.com/request-trial.html.
