# Author Correction: Clinical impact of drug-drug interactions on abemaciclib in the real-world experience of AB-ITALY study

**DOI:** 10.1038/s41523-024-00682-y

**Published:** 2024-08-02

**Authors:** Simone Scagnoli, Simona Pisegna, Angela Toss, Roberta Caputo, Michelino De Laurentiis, Michela Palleschi, Ugo de Giorgi, Enrico Cortesi, Agnese Fabbri, Alessandra Fabi, Ida Paris, Armando Orlandi, Giuseppe Curigliano, Carmen Criscitiello, Ornella Garrone, Gianluca Tomasello, Giuliana D’Auria, Patrizia Vici, Enrico Ricevuto, Federica Domati, Claudia Piombino, Sara Parola, Roberta Scafetta, Alessio Cirillo, Beatrice Taurelli Salimbeni, Francesca Sofia Di Lisa, Lidia Strigari, Robert Preissner, Maurizio Simmaco, Daniele Santini, Paolo Marchetti, Andrea Botticelli

**Affiliations:** 1 Department of Radiological, Oncological and Pathological Science, “Sapienza” University of Rome, Rome, Italy; 2 Department of Experimental Medicine, Sapienza University, Rome, Italy; 3 Department of Oncology and Hematology, Azienda Ospedaliero-Universitaria di Modena, Modena, Italy; 4 Department of Medical and Surgical Sciences, University of Modena and Reggio Emilia, Modena, Italy; 5 Department of Breast and Thoracic Oncology, Division of Breast Medical Oncology, Istituto di Ricovero e Cura a Carattere Scientifico (IRCCS) Pascale, Naples, Italy; 6 IRCCS Istituto Romagnolo per lo Studio dei Tumori “Dino Amadori” IRST Meldola IT, Meldola, Italy; 7 UOC Belcolle Hospital, Viterbo, Italy; 8 Precision Medicine in Senology, Department of Women Child and Public Health, Fondazione Policlinico Universitario A. Gemelli IRCCS, Rome, Italy; 9 Precision Medicine in Senology, Scientific Directorate, Fondazione Policlinico Universitario A. Gemelli IRCCS, Rome, Italy; 10 Division of Gynecologic Oncology, Department of Woman and Child Health and Public Health, Fondazione Policlinico Universitario Agostino Gemelli IRCCS, Rome, Italy; 11 Fondazione Policlinico Universitario Agostino Gemelli IRCCS Comprehensive Cancer Center, Unit of Medical Oncology, Rome, Italy; 12 Division of New Drugs and Early Drug Development for Innovative Therapies, European Institute of Oncology, IRCCS, Milan, Italy; 13 Department of Oncology and Hematology (DIPO), University of Milan, Milan, Italy; 14 Fondazione IRCCS Ca’ Granda Ospedale Maggiore Policlinico, SC Oncologia Medica, Milan, Italy; 15 Sandro Pertini Hospital Unit of Medical Oncology, Rome, Italy; 16 Istituto di Ricovero e Cura a Carattere Scientifico (IRCCS) Istituto Nazionale Tumori Regina Elena, UOSD Sperimentazioni di fase IV IT, Rome, Italy; 17 University of L’Aquila, L’Aquila, Italy; 18 Università Campus Biomedico, Rome, Italy; 19 IRCSS AOU Bologna, Bologna, Italy; 20 Institute of Physiology and Science-IT, Charité-Universitätsmedizin Berlin, Berlin, Germany; 21 Laboratory of Clinical Biochemistry, Sant’Andrea University Hospital, Rome, Italy; 22 Department of Neurosciences, Mental Health and Sensory Organs (NESMOS), Sapienza University of Rome, Rome, Italy; 23 Department of Medico-Surgical Sciences and Biotechnologies, Sapienza University of Rome, Rome, Italy; 24 Medical Oncology A, AOU Policlinico Umberto I, Rome, Italy; 25 Istituto Dermopatico dell’Immacolata IRCCS, Rome, Italy

**Keywords:** Breast cancer, Tumour biomarkers, Targeted therapies, Drug regulation

Correction to: *npj Breast Cancer* 10.1038/s41523-024-00657-z, published online 17 July 2024

In this article, the affiliation details for Giuseppe Curigliano were incorrectly given as “12Division of New Drugs and Early Drug Development for Innovative Therapies, European Institute of Oncology, IRCCS, Milan, Italy” but should have been ‘12Division of New Drugs and Early Drug Development for Innovative Therapies, European Institute of Oncology, IRCCS, Milan, Italy’, ‘13Department of Oncology and Hematology (DIPO), University of Milan, Milan, Italy’ and for, Carmen Criscitiello were incorrectly given as ‘13Department of Oncology and Hematology (DIPO), University of Milan, Milan, Italy’ but should have been ‘12Division of New Drugs and Early Drug Development for Innovative Therapies, European Institute of Oncology, IRCCS, Milan, Italy’, ‘13Department of Oncology and Hematology (DIPO), University of Milan, Milan, Italy’. The original article has been corrected.

