# Supplementary material for: Clinical impact of drug-drug interactions on abemaciclib in the real-world experience of AB-ITALY study
Source: NPJ Breast Cancer. 2024 Jul 17;10:58. doi: 10.1038/s41523-024-00657-z (PMC11254918; doi:10.1038/s41523-024-00657-z)
Supplement: Supplementary file 1 — Supplemental material [file 41523_2024_657_MOESM1_ESM.pdf]

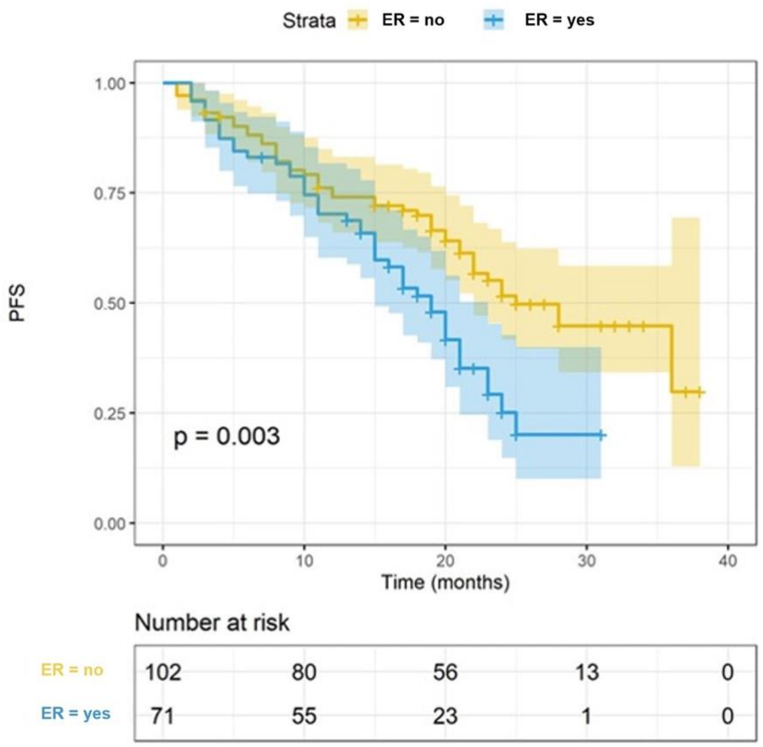

Suppl Fig 1: Shown are Kaplan–Meier estimates of PFS, according to endocrine resistance (no endocrine resistance, yellow line; endocrine resistance, blue line; p=0.003). The colored area represents the confidence interval. Tick marks represent data censored at the last time the patient was known to be alive.

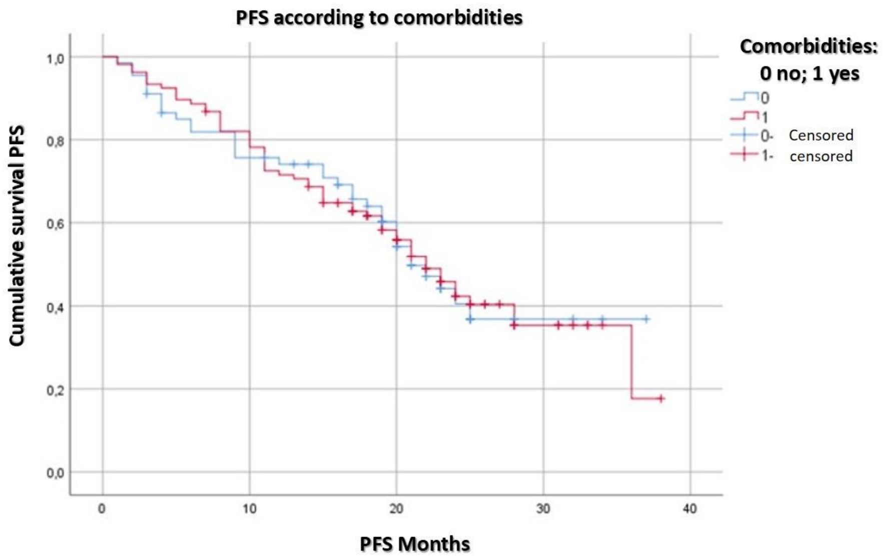

Suppl Fig 2: Shown are Kaplan–Meier estimates of PFS, according to comorbidity (no comorbidities 0, blue line; with comorbidities, red line; 22 vs 21months, p=0.998). Tick marks represent data censored at the last time the patient was known to be alive.

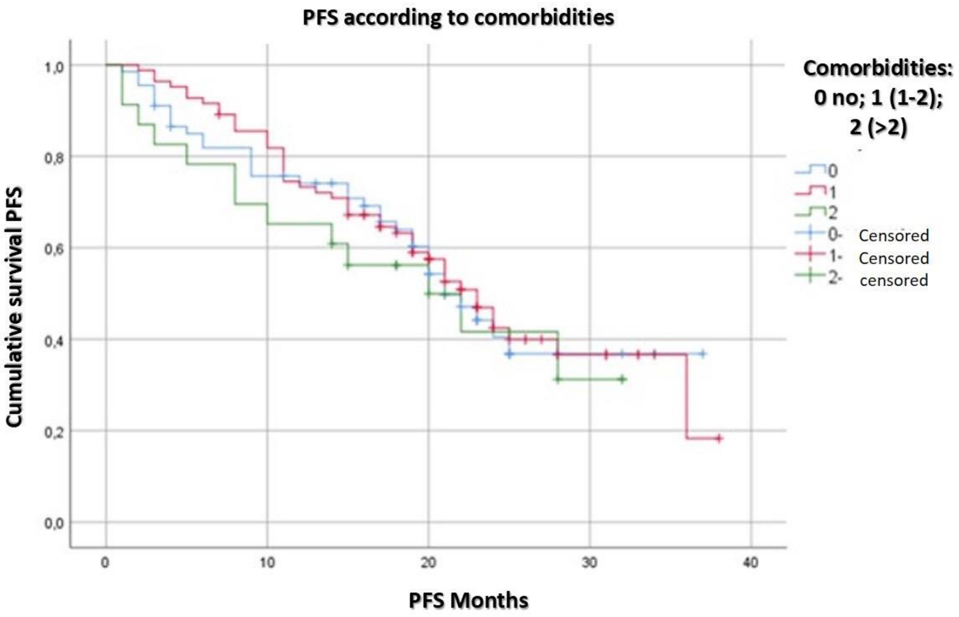

Suppl Fig 3: Shown are Kaplan–Meier estimates of PFS, according to comorbidities (no comorbidities 0, blue line; with 1 comorbidity, red line; with >1 comorbidities, green line; 22 vs 21 vs 20 months, p=0.766). Tick marks represent data censored at the last time the patient was known to be alive.

Pairwise comparison

	Age ranges	<65 Chi-square	<65 p	65-69 Chi-square	65-69 p	70-74 Chi-square	70-74 p	>75 Chi-square	>75 p
Log Rank (Mantel-Cox)	<65			2,164	,141	,953	,329	6,875	,009
	65-69	2,164	,141			,317	,574	,256	,613
	70-74	,953	,329	,317	,574			1,515	,218
	>75	6,875	,009	,256	,613	1,515	,218		

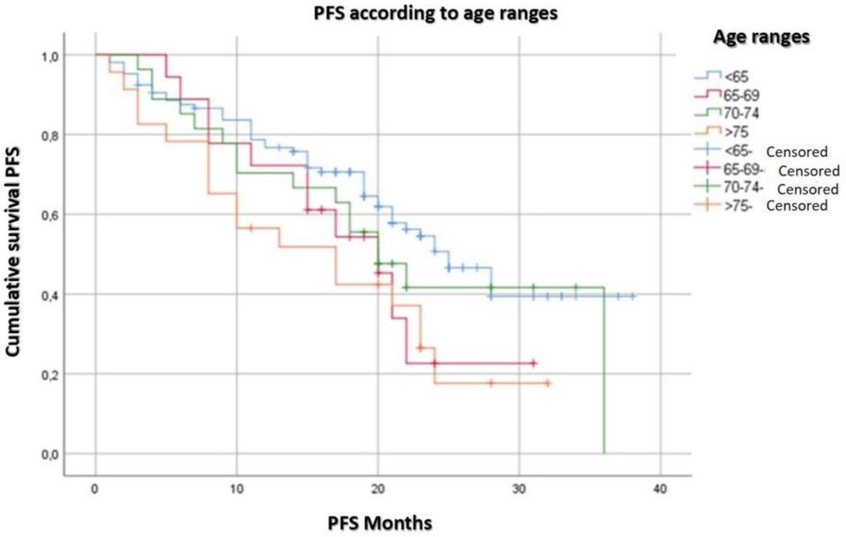

Suppl Fig 4: Shown are Kaplan–Meier estimates of PFS, according to age-based groups (<65y 0, blue line; 65-69y, red line; 70-74y, green line; >=75y, orange line; 25 vs 20 vs 20 vs 18 months; p=0.009 for <65 vs >75). Tick marks represent data censored at the last time the patient was known to be alive. Red circle highlights the significant p value.

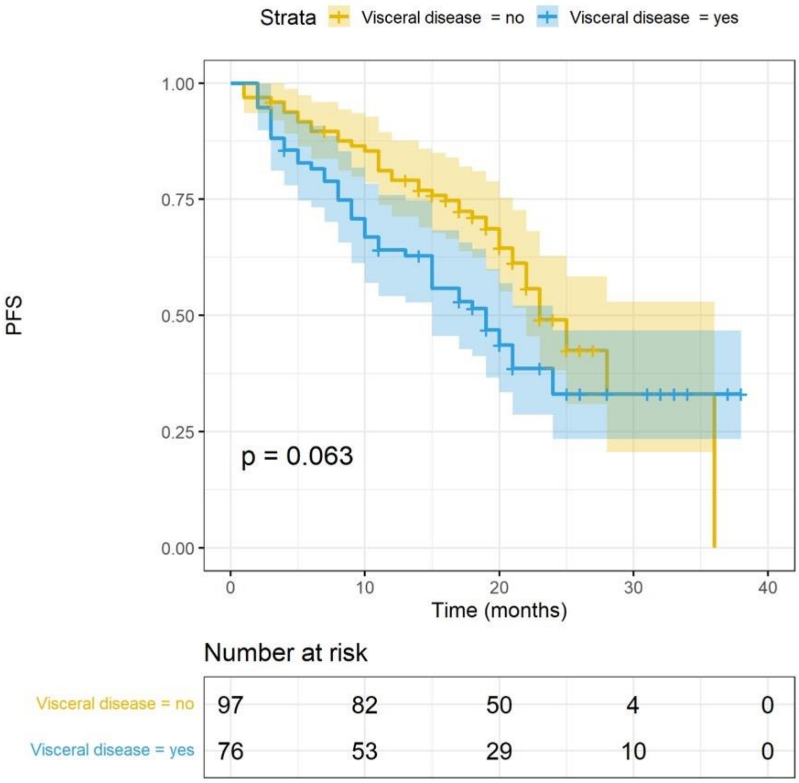

Suppl Fig 5: Shown are Kaplan–Meier estimates of PFS, according to visceral disease (no visceral disease, yellow line; visceral disease present, blue line; 23 vs. 19 months, p=0.063). The colored area represents the confidence interval. Tick marks represent data censored at the last time the patient was known to be alive.

Model	Variables included	B	Gl	p	HR	95% CI per HR Inferior	superior
Phase 1	Drug associated with abemaciclib (AI or fulvestrant)	1,150	1	0,000	3,157	1,976	5,042
Phase 2	1) Drug-PIN tier (green yellow vs other)	1,025	1	0,001	2,786	1,554	4,997
	2) Drug associated with abemaciclib (AI or fulvestrant)	1,239	1	0.000	3,452	2,145	5,556
Phase 3	1) Age (<65 or ≥65)	0,562	1	0.008	1,755	1,156	2,666
	2) Drug-PIN tier (green yellow vs other)	0,928	1	0,002	2,529	1,402	4,561
	3) Drug associated with abemaciclib (AI or fulvestrant)	1,316	1	0.000	3,727	2,304	6,030
Phase 4	1) Age (<65 or ≥65) 3) Drug associated with abemaciclib (AI or fulvestrant)	0,305	1	0.011	1,554	1,094	2,484
	2) Drug-PIN tier (green yellow vs other)	0.908	1	0.013	2,214	1,209	4,149
	3) Drug associated with abemaciclib (AI or fulvestrant)	1,402	1	0.000	3,844	2,359	6,130
	4) visceral disease (yes vs no)	0.841	1	0.048	1,981	1,478	2,934

Suppl Fig 6: shown are the results of the MVA. Sign. Indicates the p value. Exp(B) indicates the HR. The association (abemaciclib plus AI or Fulvestrant) is the first variable included; Drug-PIN tier is the second, age >65 is the third and visceral disease is the fourth.

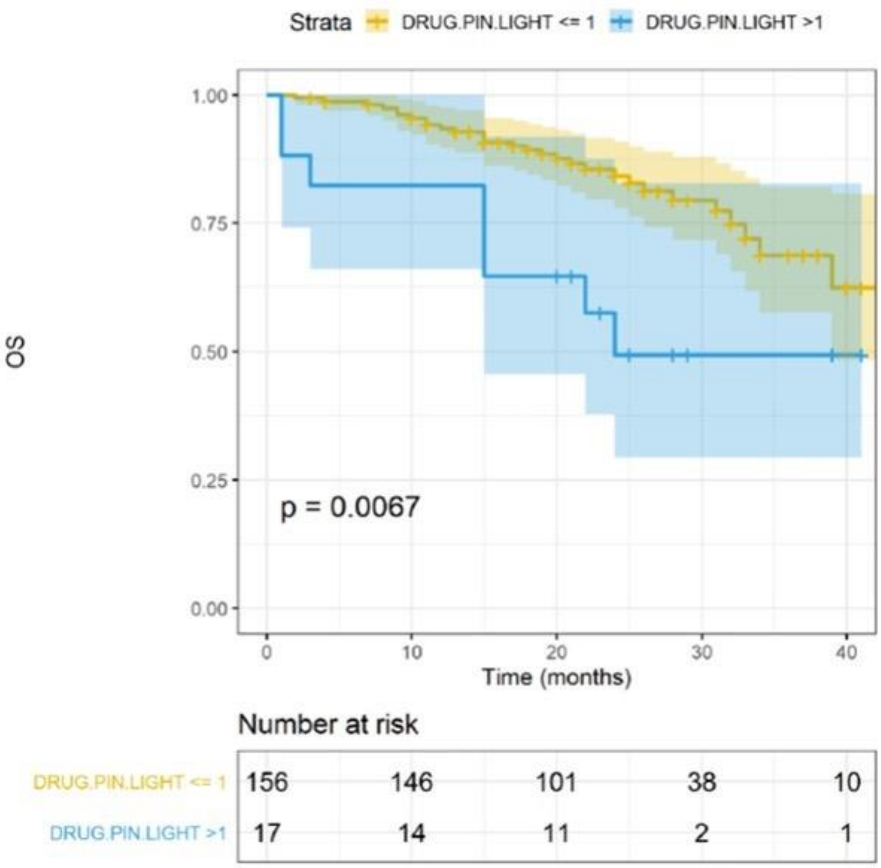

Suppl Fig 7: Shown are Kaplan–Meier estimates of OS, according to Drug-PIN light (<=1 low interactions, yellow line; >1, high interactions; not reached vs 24 months, p=0.0067). The colored area represents the confidence interval. Tick marks represent data censored at the last time the patient was known to be alive.

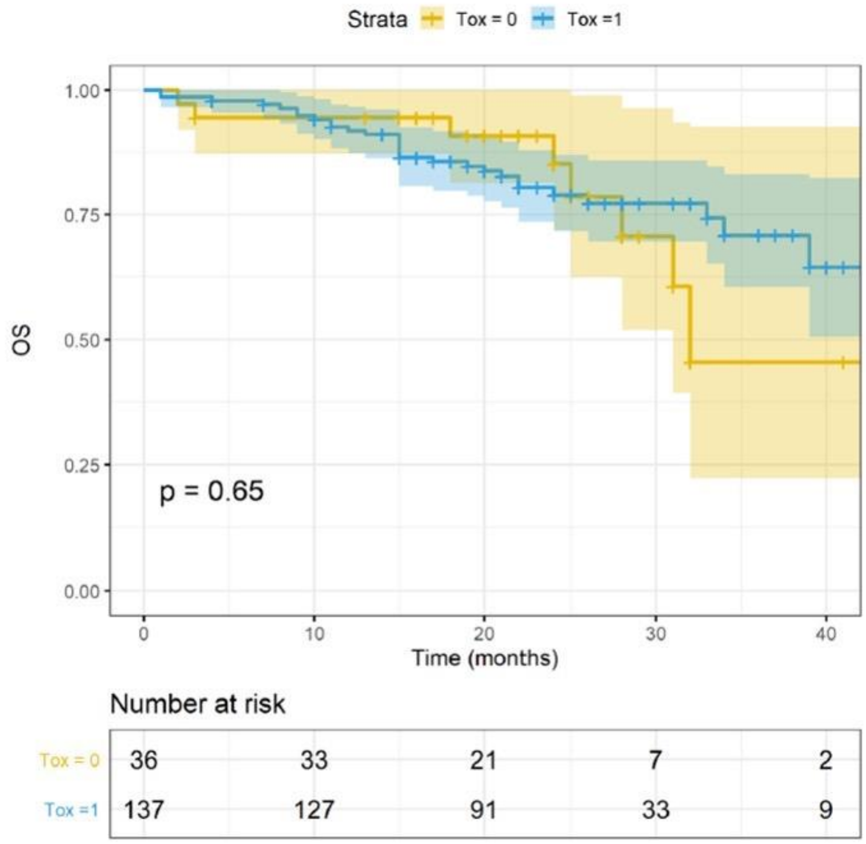

Suppl Fig 8: Shown are Kaplan–Meier estimates of OS, according to toxicity group (0=no toxicity, yellow line; 1=at least one toxicity of any grade, blue line any grade; 32 months vs not reached, p=0.65). The colored area represents the confidence interval. Tick marks represent data censored at the last time the patient was known to be alive.

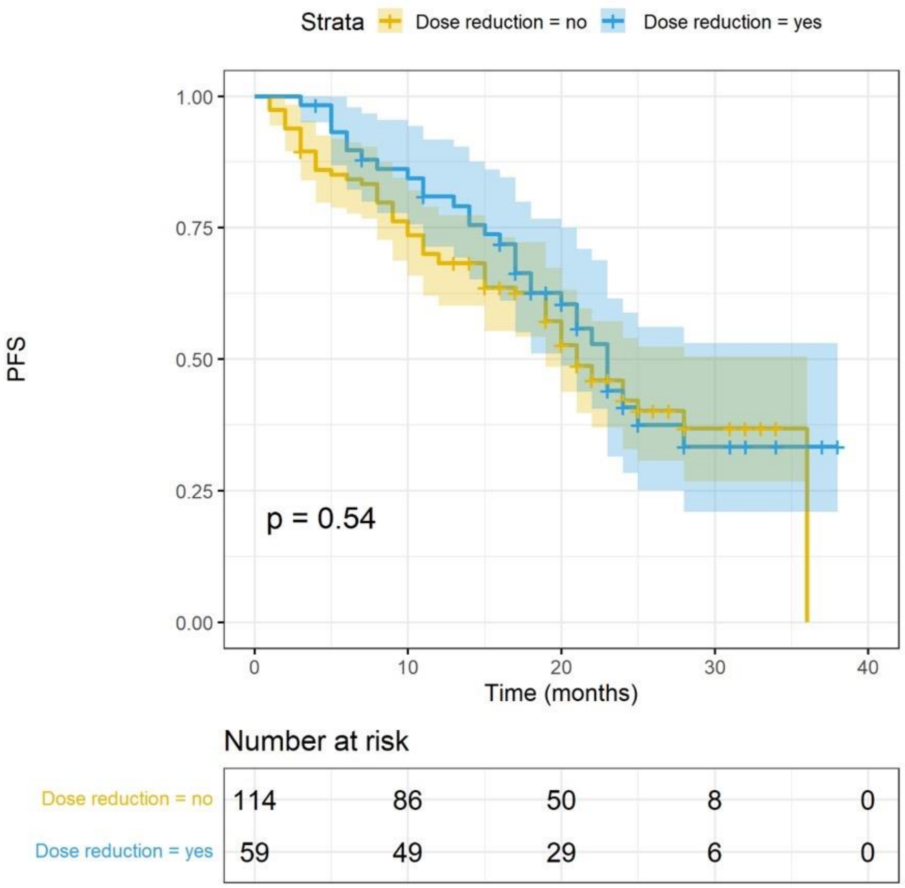

Suppl Fig 9: Shown are Kaplan–Meier estimates of PFS, according to dose reduction (no dose reduction, yellow line; dose reduction, blue line; p=0.54). The colored area represents the confidence interval. Tick marks represent data censored at the last time the patient was known to be alive.

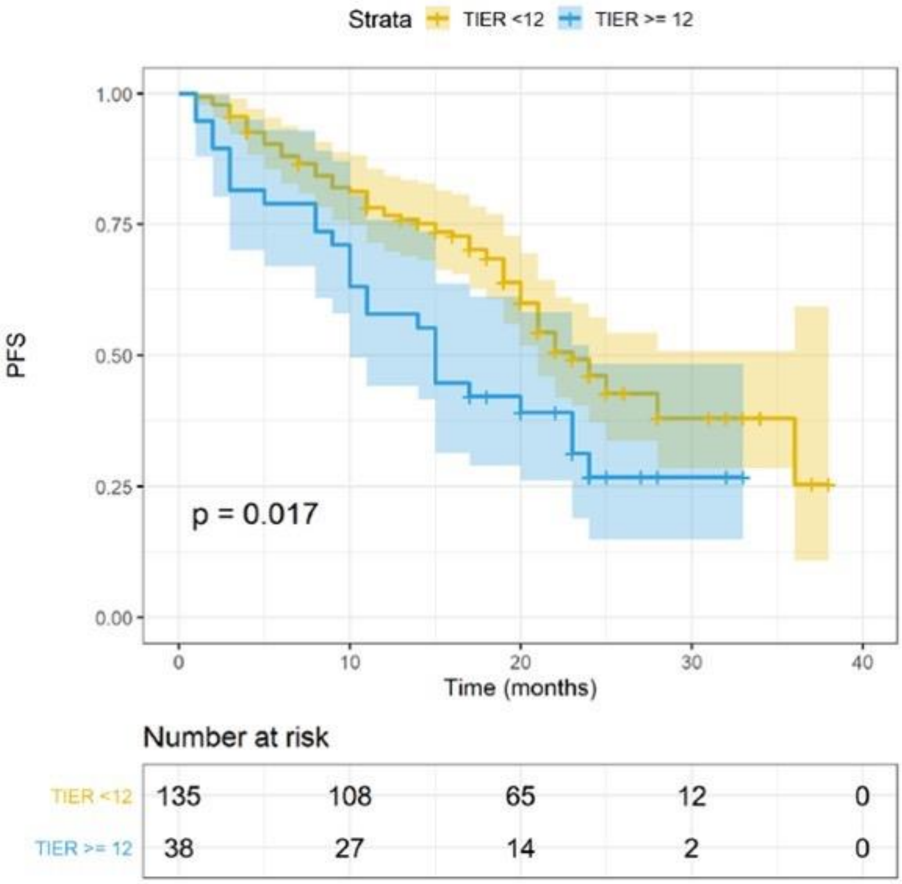

Suppl Fig 10. Shown are Kaplan–Meier estimates of PFS, according to Drug Pin score (<12, yellow line; ≥12, blue line any grade; p=0.017). The colored area represents the confidence interval. Tick marks represent data censored at the last time the patient was known to be alive.

Suppl. Table 1. Participants centres and local Ethic Committees.

Azienda Ospedaliero-Universitaria Policlinico Umberto I, Rome Italy
Azienda Ospedaliero-Universitaria di Modena, Modena, Italy
Istituto di Ricovero e Cura a Carattere Scientifico (IRCCS) Pascale, Naples, Italy
IRCCS Istituto Romagnolo per lo Studio dei Tumori "Dino Amadori" IRST Meldola IT, Meldola, Italy
Ospedale di Belcolle, Viterbo, Italy
Fondazione Policlinico Universitario A. Gemelli IRCCS, Rome, Italy.
European Institute of Oncology (IEO) IRCCS, Milan, Italy
Fondazione IRCCS Ca' Granda Ospedale Maggiore Policlinico, Milan, Italy
Ospedale Sandro Pertini, Rome, Italy
Istituto di Ricovero e Cura a Carattere Scientifico (IRCCS) Istituto Nazionale Tumori Regina Elena, Rome, Italy
Ospedale San Salvatore, L’Aquila, Italy
Fondazione Policlinico Univeristario Campus Bio Medico, Rome Italy

Suppl. Table 2. Overall response according to RECIST criteria.

**Best response**	**N (%)**
complete response (CR)	6 (4)
partial response (PR)	92 (59)
stable disease (SD)	46 (29)
progressive disease (PD)	12 (8)
ORR	98 (63)
CBR	144(92)

N: number of patients, ORR: overall response rate; CBR: clinical benefit rate

Suppl Table 3. UVA of association between Drug-PIN score or light with different AEs

variable	AEs	p-value
**Drug-PIN light**	**Diarrhea grade >= 2**	**0.07492**
**Drug-PIN light**	**DVT**	**0.09152**
Drug-PIN light	Diarrhea any grade	0.1323
Drug-PIN light	Asthenia grade >1	0.1416
Drug-PIN score	Neutropenia grade >1	0.1249
**Drug-PIN score**	**ANEMIA any grade**	**0.0675**
Drug-PIN score	DVT	0.1504

In the table are reported only the association with a p value <0.15. Association with a p value <0.1 are reported as “trends” and highlighted in bold; DVT: deep vein thrombosis

Suppl. Table 4. Collinearity evaluation for MVA of PFS

**variables**	**cVIF**
Drug-PIN tier	1.054704
Age	1.068649
ET association	1.014875
visceral disease	1.091756
